# Deep Sequencing the Transcriptome Reveals Seasonal Adaptive Mechanisms in a Hibernating Mammal

**DOI:** 10.1371/journal.pone.0027021

**Published:** 2011-10-28

**Authors:** Marshall Hampton, Richard G. Melvin, Anne H. Kendall, Brian R. Kirkpatrick, Nichole Peterson, Matthew T. Andrews

**Affiliations:** 1 Department of Biology, University of Minnesota Duluth, Duluth, Minnesota, United States of America; 2 Department of Mathematics and Statistics, University of Minnesota Duluth, Duluth, Minnesota, United States of America; 3 BioMedical Genomics Center, University of Minnesota, Saint Paul, Minnesota, United States of America; Pennington Biomedical Research Center, United States of America

## Abstract

Mammalian hibernation is a complex phenotype involving metabolic rate reduction, bradycardia, profound hypothermia, and a reliance on stored fat that allows the animal to survive for months without food in a state of suspended animation. To determine the genes responsible for this phenotype in the thirteen-lined ground squirrel (*Ictidomys tridecemlineatus*) we used the Roche 454 platform to sequence mRNA isolated at six points throughout the year from three key tissues: heart, skeletal muscle, and white adipose tissue (WAT). Deep sequencing generated approximately 3.7 million cDNA reads from 18 samples (6 time points ×3 tissues) with a mean read length of 335 bases. Of these, 3,125,337 reads were assembled into 140,703 contigs. Approximately 90% of all sequences were matched to proteins in the human UniProt database. The total number of distinct human proteins matched by ground squirrel transcripts was 13,637 for heart, 12,496 for skeletal muscle, and 14,351 for WAT. Extensive mitochondrial RNA sequences enabled a novel approach of using the transcriptome to construct the complete mitochondrial genome for *I. tridecemlineatus*. Seasonal and activity-specific changes in mRNA levels that met our stringent false discovery rate cutoff (1.0×10^−11^) were used to identify patterns of gene expression involving various aspects of the hibernation phenotype. Among these patterns are differentially expressed genes encoding heart proteins AT1A1, NAC1 and RYR2 controlling ion transport required for contraction and relaxation at low body temperatures. Abundant RNAs in skeletal muscle coding ubiquitin pathway proteins ASB2, UBC and DDB1 peak in October, suggesting an increase in muscle proteolysis. Finally, genes in WAT that encode proteins involved in lipogenesis (ACOD, FABP4) are highly expressed in August, but gradually decline in expression during the seasonal transition to lipolysis.

## Introduction

The investigation of complex phenotypes in eukaryotes has been largely confined to a handful of exhaustively studied “model organisms.” The advantage of studying this small number of plants and animals is due to a combination of factors such as ease of maintenance, a large research community, genetic selection of mutants, funded stock centers, and sequenced genomes. As a result major advances in genetics and molecular biology have been made using this select group of species. Of course, the living world is filled with novel phenotypes that are not found in the most commonly studied organisms. Mammalian hibernation is one of these phenotypes.

Hibernation is a seasonal adaptation that results in a major departure from standard mammalian homeostasis. Radical depressions in metabolism, heart rate, body temperature and oxygen consumption allow small mammals to survive up to 6 months with little or no food in a state of suspended animation called torpor (for review see [1]). Near freezing body temperatures, heart rates of 4–6 beats per minute, and depressed metabolism are characteristics of the deep torpor that occurs during hibernation. Mysteriously, hypothermic torpor bouts are interrupted by brief normothermic interbout arousals (IBAs) every 1–3 weeks depending on the species [2]. The mechanistic basis of the hibernation phenotype is unknown, but mounting evidence from a wide variety of animals suggests that it is controlled in part by the differential expression of specific genes [3], [4].

Over the past decade microarray hybridization has been commonly used as a means of measuring gene activity by whole tissue transcription profiling. More recently massive parallel sequencing has played a larger role in quantifying gene expression by deep sequencing the transcriptome. The term “deep sequencing” refers to the millions of RNA sequence reads that are generated by this method. Deep sequencing the transcriptome, also known as RNAseq, provides both the sequence and frequency of RNA molecules that are present at any particular time in a specific cell type, tissue or organ. Counting the number of mRNAs that are encoded by individual genes provides an indicator of protein-coding potential, a major contributor to phenotype.

We have performed RNAseq using the Roche 454 system to identify genes that are expressed in active and hibernating thirteen-lined ground squirrels (*Ictidomys tridecemlineatus)* at six points throughout the year. We used this approach to obtain a better understanding of the hibernation phenotype in three distinct organs: heart, skeletal muscle, and white adipose. The heart is a contractile organ that must continue to work despite the physiological extremes of torpor. Skeletal muscle is inactive when the animal is in torpor and sees only limited activity during periodic IBAs. White adipose tissue is the primary fuel storage depot for both organs throughout the hibernation season.

Deep sequencing the transcriptome offers many advantages over other methods for measuring gene activity such as microarrays, which have hybridization bias detection, higher background and require more sample quantity. The 454 platform generates individual RNA sequence reads up to 600 bases in length. This size is useful for non-model organisms such as the thirteen-lined ground squirrel where longer read length greatly facilitates reliable assembly and identification of the corresponding transcript. In this study we generated 3.7 million reads, added original cDNA sequence to the thirteen-lined ground squirrel database, and report a novel approach for sequencing the mitochondrial genome that can be applied to other eukaryotes. Moreover, we have uncovered new patterns of differential gene activity throughout the year that contribute to our understanding of the hibernation phenotype.

## Materials and Methods

### Animals

All animal use in this study was carried out in strict accordance with the approval of the University of Minnesota Institutional Animal Care and Use Committee (protocol #0805A34502). Thirteen-lined ground squirrels were wild-caught in central Minnesota. Animals were housed at the University of Minnesota School of Medicine Duluth, where they were fed Purina Laboratory Rodent Diet 5001 and provided with water *ad libitum*. Animals were maintained at 23°C with a 12∶12 light:dark cycle (7 a.m. to 7 p.m.).

For each time point, 3 males and 3 females with weights within one standard deviation of the mean were selected for study. Animals with obvious health defects were excluded from consideration. A summary of animal characteristics including weight, body temperature, and food availability at the time of sacrifice is provided in Table 1. April animals were sacrificed within 48 hours of capture in the wild to measure gene expression in the early spring when active animals are leanest. August active animals were in the fattening phase and continued to gain weight in preparation for hibernation. Active animals in October reached a plateau in body weight and most animals began to enter brief torpor bouts in captivity despite continued ambient conditions of 12L∶12D at 23°C [5]. On October 30 animals captured in the previous spring and summer were switched to 24 hour darkness at 5°C to mimic the natural conditions of underground burrows during the hibernation season. January torpor and IBA animals were sacrificed after two months of uninterrupted darkness, ambient temperature of 5°C, and no food. On March 12 light and temperature returned to 12L∶12D at 23°C. Aroused March animals were maintained for 24 hours without food after returning to non-hibernating conditions of light and temperature. Active animals sacrificed for this final time point therefore spent the previous 4.5 months under fasting conditions.

**Table 1:** Time points (months) and corresponding states of activity, mean animal mass, mean body temperature, and food availability at the time when tissues were collected for RNA extraction.

Collection point	State	Mass	Body Temperature	Food Availability
April	Active	165±14 g	37.6±0.4°C	Yes
August	Active	231±9 g	36.7±0.4°C	Yes
October	Active	248±10 g	32.4±1.9°C	Yes
January	Torpid	176±13 g	6.0±0.4°C	No
January	IBA	180±10 g	28.1±3.0°C	No
March	Arousal	163±25 g	35.3±0.4°C	No

*Abbreviation* – IBA, interbout arousal.

### Tissue collection

All animal surgical procedures were performed under isoflurane anesthesia and all efforts were made to minimize potential pain and suffering. For each of the 6 collection points throughout the year, all animals were sacrificed during the same 9 a.m. to 4 p.m. time frame. Heart, skeletal muscle, and white adipose tissues were collected on ice immediately after sacrifice. The heart was separated from the pericardium, brown fat and attached vessels. Skeletal muscle was collected from the hind limb vastus lateralis. Visceral white adipose tissue (WAT) was dissected to collect only the retroperitoneal fat pad [6]. The retroperitoneal fat pad of April animals was very small; therefore to obtain enough WAT for RNA preparation the mesenteric fat pad was included in some samples. Inclusion of mesenteric WAT may have introduced some contamination with pancreatic tissue [7] in the April WAT samples. All tissues were flash frozen in liquid nitrogen and stored at –80°C until processed for total RNA isolation.

### Total RNA isolation

Total RNA was isolated from 250 mg each of heart, skeletal and white adipose tissue. Briefly, 250 mg of frozen tissue was thawed in 1 mL of ice cold TriReagent (Ambion/Applied Biosystems, Foster City, CA) and homogenized on ice using a Tissue Tearor homogenizer (BioSpec Products Inc., Bartlesville, OK). The homogenates were centrifuged at 12,000× g, 4°C for 10 min using a Beckman F2402H rotor and Avanti centrifuge (Beckman Coulter, Inc. Brea, CA) to pellet cell debris. For WAT only, the cell lysate was recovered from beneath the upper lipid layer, combined with 1 volume TriReagent and centrifuged a second time (12,000×g, °C, 10 min). Cell lysates were incubated 5 min at room temperature, and combined with 1-bromo-3-chloropropane (Sigma, St. Louis, MO). Aqueous and organic phases were separated by centrifugation (12,000× g, 4°C, 15 min) and RNA was extracted using Qiagen RNeasy mini column protocol for RNA isolation from animal tissues (Qiagen, Valencia, CA).

Quality of the isolated total RNA was assessed by non-denaturing agarose gel electrophoresis (1.5% agarose, 1× Tris-acetate-EDTA buffer, 0.05 µg/ mL ethidium bromide) and quantity of RNA was determined using Nanodrop spectrophotometer (Thermo Fischer Scientific Inc., Waltham, MA). Potential contaminating DNA was removed by treatment with DNase 1 using an Ambion Turbo DNA*free* kit (Ambion/Applied Biosystems, Foster City, CA). Total RNA from individual animals were used in qRT-PCR experiments (Figure S2). Equal amounts of total RNA from individual animals at the same time points (3 females, 3 males) were pooled for poly (A)+ RNA purification and subsequent 454 sequencing.

### Poly(A)+ RNA purification

The following poly(A)+ RNA isolation, library preparation, emPCR and sequencing was carried out by the BioMedical Genomics Center at the University of Minnesota. Ethanol precipitation of total ground squirrel RNA preparations was performed. The RNA pellet was rehydrated in 100 µL of nuclease-free water. All samples were run on an Agilent 6000 Nano chip on the BioAnalyzer 2100 to verify the integrity of the RNA. All samples had a BioAnalyzer RIN number of 8 or greater, signifying high quality RNA. Poly(A)+ RNA was prepared from 10 µg of total RNA in a total of 250 µL of nuclease-free water and added 250 µL of 2× Binding Solution from the Applied BioSystems MicroPoly(A)Purist Kit. We followed the manufacturer's protocol through recovery of the poly(A)+ RNA, followed by a second round of oligo (dT) selection.

### cDNA rapid library preparation using Multiplex Identifiers (MIDs)

Preparing the cDNA library consisted of 9 major steps: 1) fragmentation of RNA, 2) double-stranded cDNA synthesis, 3) fragment end repair, 4) AMPure Bead preparation, 5) rapid library MID adaptor ligation, 6) small fragment removal, 7) library quantification, 8) cDNA library quality assessment, and 9) preparation of working aliquots. Solutions prepared prior to starting the protocol were 10 mM Tris-HCL pH 7.5, 70% ethanol, RNA fragmentation solution, 0.2 M EDTA pH 8.0, and 400 µM Roche Primer “random”. All of the samples were prepared using the individual sample clean-up (ISC) method (Section 3.4 and 3.6 of the Roche cDNA Rapid Library Preparation Method Manual, October 2009). Each of the individual samples was indexed using one of the twelve Roche MID adaptors. We used the TBS-380 from Turner BioSystems to quantify the DNA library (Section 3.7.2 of the Roche cDNA Rapid Library Preparation Method Manual, October 2009). The Rapid Library quantification calculator at www.454.com/my454 was used to generate a Rapid Library Standard Curve and to quantify each cDNA library in molecules/µl. Each library was run on a High Sensitivity chip on the BioAnalyzer 2100 to validate that the library size was between 600–1200 base pairs and all manufacturer protocols were followed. A working stock of 1×10^7^ molecules/µl in TE buffer was made for each library.

### Emulsion titration of cDNA libraries

After normalization of the individual libraries, groups of three indexed samples were pooled together in equal molar ratios and titrated to optimize yield and sequence quality. This was done using emulsion-based clonal amplification (emPCR) following the procedure outlined in the Roche emPCR Method Manual Lib-L SV, October 2009 (version 2). For each pooled cDNA library, four single tube emPCR amplification reactions were prepared using different input amounts (2, 4, 8 and 16 molecules per bead; Roche Technical Bulletin for Updated Titration Range for GS-FLX Titanium emPCR Lib-L Kit, March 2010). The TissueLyser II from Qiagen was used to prepare the emulsions prior to amplification. Amplification was carried out using a Tetrad 2 thermal cycler from BioRad. The emulsions were broken and the DNA capture beads were collected and pooled, each pool was then enriched for beads carrying single stranded cDNA (sstcDNA) according to the manufacturer's instructions (Roche). The enriched bead samples were then counted using a Z1 Coulter Counter (Beckmann Coulter) to calculate the percent enrichment (i.e., the percent of initial beads that contained sstcDNA). Based on a linear regression of these percent enrichment values against the initial sstcDNA amounts, we calculated the amount of cDNA needed to produce an expected 8% enrichment.

### Bulk emPCR

Large volume bulk emPCR was performed as described in the emPCR Method Manual – Lib_L LV, October 2009, from Roche Applied Science. We started with two pools consisting of three indexed libraries each. For each pooled library, the copy per bead that achieved closest to 8% enrichment during the titration was used for bulk emPCR. Two large volume emulsification reactions were prepared for each pooled library to be loaded on a 2-region PicoTiterPlate (PTP) from Roche Applied Science. Roche's vacuum-assisted protocol for emulsion breaking and bead recovery for large volume emulsions was followed. Bulk library pools that resulted in 8-15% enrichment were used for sequencing.

### Titanium sequencing

Sequencing of each pooled library was carried out on the Genome Analyzer FLX. The PTP was divided using a 2-region gasket. The number of DNA library beads loaded per region was approximately 2 million. The Roche GS GLX Titanium Sequencing and PicoTiterPlate Kits were used to conduct three full sequencing runs. Each of the three runs contained two pools of three different indexed libraries. Image analysis and base-calling software were performed with standard protocols and default parameters. Each indexed library represented between 11–28% of the total number of reads.

### Bioinformatics

**Figure 1:**
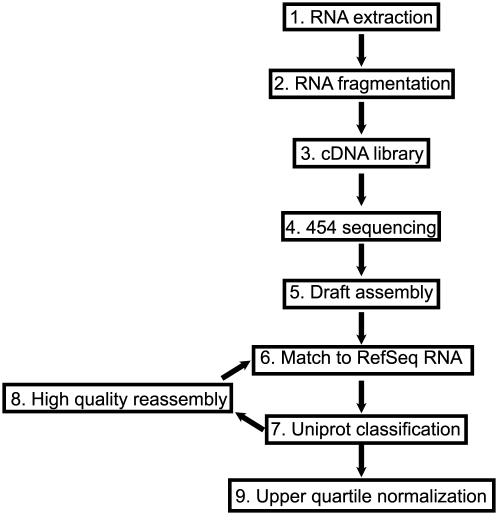
Summary of sample preparation and the bioinformatic pipeline used in this study.

The bioinformatic pipeline for converting raw reads into identified sequence is summarized in Figure 1. Roche Multiplex Identifier oligonucleotides (MIDs) ligated to cDNA ends were used to sort each of the 18 samples. To remove possible MIDs, and potential PCR adaptor sequences, 26 bases were trimmed from every read. The open source program MIRA [8] assembled the reads into contigs (Figure 1, step 5). The MIRA option string used for the creation of final contigs was: “--job = denovo,est,normal,454 454_SETTINGS -CL:emlc = yes -CL:mlcr = 16 -CL:emrc = yes -CL:mrcr = 20”. Our contigs and singleton reads were combined into a nonredundant set of sequences which were then identified versus the human mRNA RefSeq database from NCBI (release 45) using the NCBI Blast+ suite (version 2.2.24) program BLASTn [9] with an expectation value cutoff of 10^−5^ (Figure 1, step 6). The BLAST results were mapped to the human UniProt database [10] to identify the protein-coding potential for all 18 samples. Finally, each set of reads that were mapped to a single UniProt protein was re-assembled with MIRA using the "accurate" option.

To compare the RNA level of individual genes across all six collection points we used upper quartile normalization as described in the supplementary materials of Bullard et al. [11]. We interpret normalized transcript levels, the number of times a gene sequence is counted in a sample normalized to all other samples within the same tissue, as the mRNA expression of that gene. We do not correct for the effect of transcript length (longer transcripts will be over represented) because we could not consistently determine those lengths without a reference genome, so expression levels can only be reliably compared across time points within a single gene and tissue. An important additional source of error arises from artificial sequence replicates during the PCR emulsion step of the 454 amplification and sequencing, when more than one streptavidin bead is present in a PCR reaction droplet [12]. In such cases, many identical reads can be generated, distorting the expression signature of a gene. We corrected for these replicates by removing reads that were identical in the first 36 bases when at least three such reads were present at only one time point.

To identify differentially expressed genes across the six sampled points we first determined the p-value of a six-way Fisher's exact test for each gene. These p-values were corrected for multiple comparisons by calculating false discovery rate (FDR; [13]). To create a list of differentially expressed genes of high confidence for further analysis, we used a stringent false discovery rate cutoff of 1.0×10^−11^.

## Results

### Experimental design

This study was designed to provide a yearlong examination of gene activity responsible for the hibernation phenotype. Table 1 shows the six time points when heart, skeletal muscle and white adipose tissue were harvested for the purpose of RNA preparation. The six points were chosen to provide comprehensive coverage of circannual events that contribute to the hibernation phenotype. Approximately 3.7 million cDNA sequence reads from 18 samples (6 time points ×3 tissues) were generated in 3 separate sequencing reactions. The sff (standard flowgram format) quality and sequence files from the 454 sequencing were deposited into the NCBI Short Read Archive with accession number SRA037446. The bioinformatic pipeline for converting these raw reads into identified sequence is summarized in Figure 1.

### Sequence reads and contig assembly

**Figure 2:**
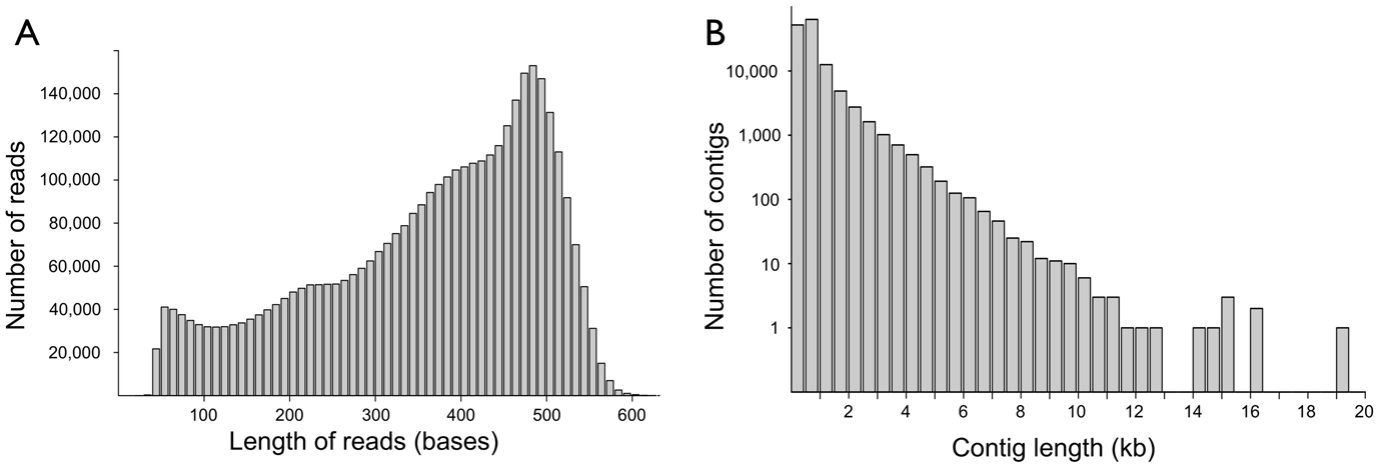
Length of raw cDNA sequence reads and contigs. **A.** Histogram of read lengths. Bins are 10 bases wide. **B.** Histogram of contig lengths. Bins are 500 bases wide. In both A and B, bars are shrunk to half size to add separation for clarity.

Figure 2A shows the size distribution of the raw sequence reads. After removing MIDs and adaptor sequences the mean trimmed read length was 335 nucleotides, with a standard deviation of 133 nucleotides. Of these reads, 3,125,337 were assembled into 140,703 contigs. Many of the finished contigs were approximately the size of the respective full-length cDNAs from the human mRNA Refseq database. Figure 2B is a histogram showing the range in contig size up to 19 Kb. As expected, overall contig abundance declined with increasing size.

The assembled contigs and remaining 619,767 unassembled sequences were used to identify proteins that are encoded by the raw sequence reads. Tables 2, 3, 4 show the RNA sequence read number for all six points in heart (Table 2), skeletal muscle (Table 3) and white adipose tissue (Table 4). Approximately 90% of all the RNA sequence reads from heart and skeletal muscle were identified and matched to sequences in the human UniProt database, with only slightly lower percentages for read identification in white adipose tissue. Interestingly the mitochondrial genome encoded approximately 21% of the heart RNAs, 10% of the skeletal muscle RNAs, but only 2% of the WAT transcripts. The total number of human UniProt proteins matched by ground squirrel transcripts was 13,637 for heart, 12,496 for skeletal muscle, and 14,351 for white adipose tissue.

**Table 2:** Heart read measurements.

Time point	August Active	October Active	January Torpor	January IBA	March Active	April Active
Total reads	424,124	259,936	328,909	217,662	176,694	270,109
RefSeq	93.1%	90.9%	94.4%	93.8%	94.7%	94.7%
UniProt identified	90.2%	87.0%	91.8%	90.4%	92.1%	91.6%
Mitochondrial	19.1%	24.7%	22.2%	17.6%	23.2%	23.8%

"RefSeq" refers to reads identified in the human mRNA RefSeq database. “UniProt identified” refers to reads identified in the human UniProt database [10]. “Mitochondrial” refers to RNAs encoded by the mitochondrial genome. *Abbreviation* – IBA, interbout arousal.

**Table 3:** Skeletal muscle read measurements.

Time point	August Active	October Active	January Torpor	January IBA	March Active	April Active
Total reads	173,086	110,748	150,778	86,220	291,697	195,567
RefSeq	95.4%	94.6%	94.5%	93.8%	94.5%	95.3%
UniProt identified	92.1%	92.2%	92.2%	91.0%	90.9%	91.6%
Mitochondrial	11.4%	6.8%	6.8%	7.2%	10.8%	18.8%

"RefSeq" refers to reads identified in the human mRNA RefSeq database. “UniProt identified” refers to reads identified in the human UniProt database [10]. “Mitochondrial” refers to RNAs encoded by the mitochondrial genome. *Abbreviation* – IBA, interbout arousal.

**Table 4:** White adipose tissue read measurements.

Time point	August Active	October Active	January Torpor	January IBA	March Active	April Active
Total reads	163,906	123,722	89,393	200,889	245,839	110,125
RefSeq	91.5%	92.1%	90.8%	91.1%	89.2%	86.5%
UniProt identified	89.2%	89.7%	88.7%	88.9%	86.8%	83.3%
Mitochondrial	3.0%	1.4%	1.0%	1.2%	1.7%	3.6%

"RefSeq" refers to reads identified in the human mRNA RefSeq database. “UniProt identified” refers to reads identified in the human UniProt database [10]. “Mitochondrial” refers to RNAs encoded by the mitochondrial genome. *Abbreviation* – IBA, interbout arousal.

### Non-protein coding RNA and MALAT1

Reads and contigs which did not match human UniProt records included ribosomal RNA and other non-protein coding RNA. The ribosomal RNA was not surprising given its abundance, despite two rounds of oligo-dT selection. A comparison (using BLASTn) of our sequences with the fRNAdb *Mus musculus* noncoding RNA database gave numerous matches [14]. Unfortunately, the vast majority of these records were unannotated. A striking exception was the sequence for MALAT1 (also known as hepcarcin), a long (6 to 7 kb) noncoding RNA that is highly conserved in mammals and known to be involved in the regulation of RNA splicing [15]. The expression of MALAT1 appeared to be quite variable, with the highest levels for heart and WAT seen in October (Table 5).

**Table 5:** MALAT1 expression.

	April	August	October	Torpor	IBA	March
Skeletal muscle	1883	943	1724	1961	1708	799
Heart	2849	2834	5056	2345	1355	3039
WAT	635	388	1636	412	448	367

Upper-quartile normalized counts for reads matching the *Mus musculus* MALAT1/hepcarcin long non-protein coding RNA.

### Sequence read density

The assembly of multiple raw sequence reads for a single mRNA generates high quality sequence due to the redundancy of reads spread throughout the contig. Multiple reads across the same region of the transcript increases sequence accuracy because sequencing errors can easily be identified and corrected. The overall distribution of reads for a single mRNA typically showed a higher density near the middle of the transcript. This distribution is expected if fragmentation and random priming are unbiased simply from the probability of overlaps along the transcript (a more mathematically detailed description is given in the next section).

**Figure 3:**
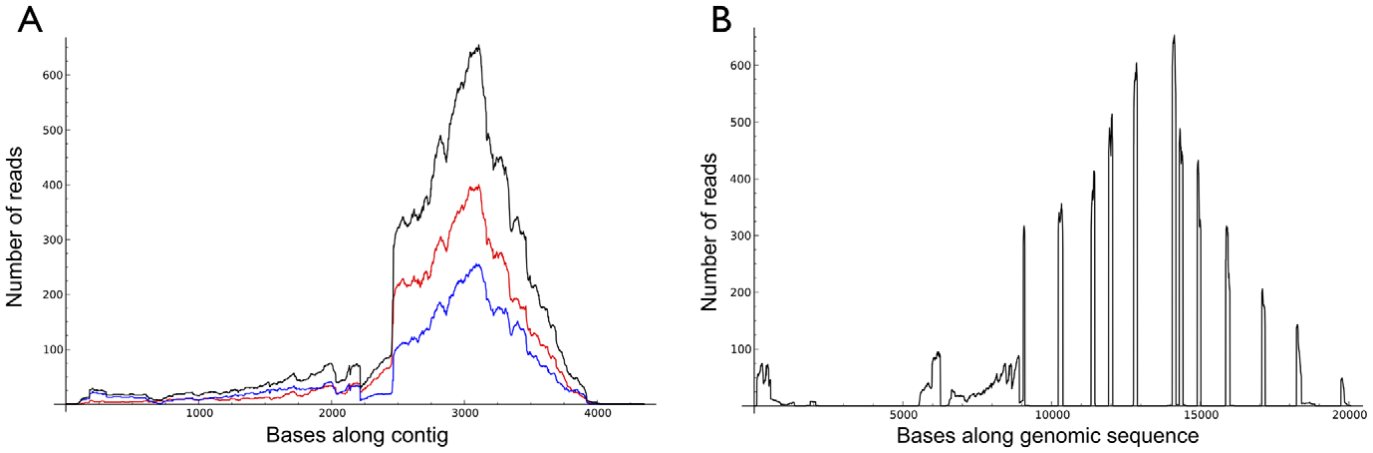
Example of sequence read density. **A**. Density of reads that align to the longest contig for the pancreatic triacylglycerol lipase (LIPP) mRNA. Total read density is in black, reads from all heart samples are in red, and reads from all white adipose tissue are in blue. **B**. The same reads aligned to a genomic contig encoding the LIPP mRNA. Note that reads are confined to exonic regions. The genomic contig is combined from the NCBI Nucleotide record AY071823 [21] and the Ensembl speTri1 assembly GeneScaffold 5826, bases 51570-63355.

To demonstrate sequence read coverage, Figure 3A shows the read density from two tissues over the largest contig encoding pancreatic triacylglycerol lipase (LIPP). LIPP is normally expressed in the pancreas, but during hibernation it provides low temperature lipolysis in both heart and white adipose tissue [16], [17], [18]. The figure shows the majority of the reads are near the center of the coding region with fewer near the ends, including the 5′ UTR sequences which differ between the two tissues. This same trend was seen when the reads were projected over the much longer LIPP genomic sequence (Figure 3B). As predicted, the reads were confined to the exonic sequences of the LIPP gene as shown by the distinct spikes in read number. Due to the current 2× sequence coverage of the *I. tridecemlineatus* genome, Figure 3B is not complete because the 3′ portion consists of six contigs that were assembled into a scaffold aligned against the human genome.

### Assembly of ground squirrel mitochondrial genome from RNA reads

The mitochondrial genome is transcribed as a single transcript that is then processed into smaller pieces that are polyadenylated [19]. The large number of mitochondrial reads obtained from heart and skeletal muscle enabled us to construct the complete *I. tridecemlineatus* mitochondrial genome. The vast majority of reads are from the protein coding domain of a gene, but in rare cases reads spanned two genes or included rRNA, tRNA, and the D-loop sequence. These rare cases may be from fragments of the original full-length transcript. An apparently complete mitochondrial genome sequence was constructed from four of the largest contigs produced by MIRA (Figure S1). The assembled genome sequence is 16,459 bases long, which is very similar to that of the Eurasian red squirrel (*Sciuris vulgaris*) - the closest matching sequence in Genbank, which is 16,507 bases long (NCBI accession NC_002369). All of the standard mammalian genes - protein-coding, tRNA, and rRNA - are present and complete.

### Modeling read density

The relative simplicity of mitochondrial transcripts - no introns or alternative splicing - provided a unique opportunity to examine potential biases in our library preparation. To do this we developed a mathematical model of read density.

**Figure 4:**
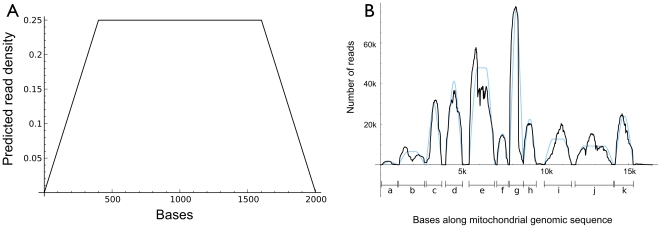
Construction of mitochondrial genome using transcriptome data. **A.** Example of the predicted density of reads along a contig 2000 bases long using a single read length of 400 bases. **B.** Actual read density (black) and model predictions (blue) for the mitochondrial transcriptome. Predictions are for (a) 12S ribosomal RNA, (b) 16S ribosomal RNA, (c) NU1M - NADH-ubiquinone oxidoreductase chain 1, (d) NU2M - NADH-ubiquinone oxidoreductase chain 2, (e) COX1 - cytochrome c oxidase subunit 1, (f) COX2 - cytochrome c oxidase subunit 2, (g) ATP6, ATP8 - ATP synthase subunit a and ATP synthase protein 8, (h) COX3 - cytochrome c oxidase subunit 3, (i) NU4M, NU4LM - NADH-ubiquinone oxidoreductase chain 4 and NADH-ubiquinone oxidoreductase chain 4L, (j) NU5M, NU6M - NADH-ubiquinone oxidoreductase chain 5 and NADH-ubiquinone oxidoreductase chain 6, (k) CYB - cytochrome b.

To model the distribution of reads along a mitochondrial gene, we assume: (1) the gene produces a single transcript type of length L, and (2) the observed distribution of read lengths results from a uniform (unbiased) fragmentation of the original transcripts. If we have a read of length J≤L drawn from this distribution, it has L-J+1 possible starting locations along the transcript. For the I^th^ position on the transcript, the number of such read locations that overlap it is the minimum of {I, J, L-I+1, L-J+1}. So the probability p(I,J,L) of a read of length J overlapping position I in a transcript of length L is min({I, J, L-I+1, L-J+1})/(L-J+1). For fixed L and J this is a piecewise-linear function of I. An example graph of this function is shown in Figure 4A for the case L = 2000 and J = 400. We average these probabilities over all the observed read lengths for each mitochondrial gene, weighted by the frequency of those lengths, which results in an expected density that is somewhat smoother than the piecewise linear components. The predicted distributions from this model are shown in blue along with the actual read distributions (in black) in Figure 4B. Coefficients of determination, R^2^, were computed for each protein-coding sequence and ranged from a high of 0.89 for COX2 to 0.05 for COX1. Deviations from the model are expected in some cases because overlapping genes do not satisfy the assumptions of the model. This does not explain the poor performance of the model for COX1, which has an anomalously low R^2^ among the sequences that contain only one protein product.

### Duplication of the ACOD gene

**Figure 5:**
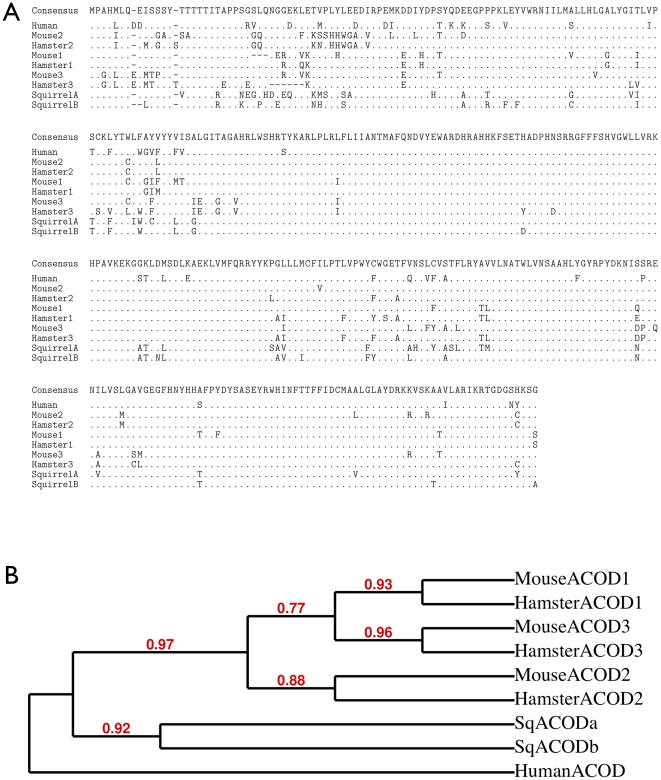
Duplication of acyl-CoA desaturase genes (ACODs). **A.** Alignment of ACODs (Acyl-CoA Desaturases) from the human, mouse, golden hamster (accessions AF097514_1, NP_033154.2, NP_033153.2, AAI18046.1, ABS71850.1, ABS71851.1, Q64420.1) and the 13-lined ground squirrel (translated from our contigs), produced by MUSCLE [58] and Gblocks [59]. Only differences from the consensus are shown. **B.** Cladogram of the ACOD sequences, using PhyML 3.0 [60] with the WAG protein scoring matrix [61] and 100 bootstrap replicates [62].

The nuclear encoded acyl-CoA desaturase (ACOD) mRNA is the single most abundant RNA transcript in white adipose tissue. ACOD converts a single carbon-carbon bond into a double bond thus turning a saturated fatty acid into an unsaturated fatty acid. This is an important reaction for a hibernating species because fatty acids with double bonds in the cis conformation have lower melting temperatures and thus greater fluidity. Examination of the thousands of ACOD reads reveals that the genome of *I. tridecemlineatus* encodes two distinct ACOD mRNAs. An alignment of the protein translations of these sequences with desaturases from human, mouse (*Mus musculus*), and golden hamster (*Mesocricetus auratus*), plus a corresponding maximum likelihood tree suggest that the *I. tridecemlineatus* ACOD gene locus was duplicated in a separate event from the Muroidea triplication (Figure 5). Separating the normalized counts for reads mapping to ACOD into these two distinct genes shows that one is much more highly expressed, but both are differentially expressed within white adipose (Table 6). It is likely that other duplicated genes are present, but high read counts, such as those seen with ACOD, are necessary for reliable identification of duplicated sequences.

**Table 6:** ACOD Expression.

Skeletal muscle						
	April	August	October	Torpor	IBA	March
ACODA	4	29	11	3	1	1
ACODB	2	6	2	11	7	2
Total	7	38	14	14	12	4

Heart						
	April	August	October	Torpor	IBA	March
ACODA	4	40	6	3	2	3
ACODB	22	18	23	18	20	3
Total	29	62	29	23	25	10

White adipose						
	April	August	October	Torpor	IBA	March
ACODA	1100	6687	5083	3690	3285	2097
ACODB	28	1361	1267	768	796	368
Total	1137	8313	7387	4561	4199	2560

Upper-quartile normalized counts for reads matching two distinct ground squirrel ACOD sequences, and the total normalized counts that were mapped to ACOD_HUMAN. Because of rounding the upper-quartile counts, and the fact that some contigs from the untranslated parts of the transcripts cannot be definitively assigned, the total counts are not always the sum of the counts of the two separate genes.

### Differential gene expression

The fundamental physiological adaptation present in natural hibernators is a greatly reduced metabolic rate with accompanying reductions in body temperature, heart rate and respiration. In this study, changes in mRNA levels of specific genes throughout the year were analyzed to identify biological processes that are likely to be important for maintaining organ function during these physiological extremes.

To validate our transcriptome approach of measuring differential gene expression we performed qRT-PCR measurements of several genes across the year (Figure S2). We tested the expression of 24 different genes in WAT isolated from individual animals (N = 6) at five time points from August through March for correlation of quantitative RT-PCR results with the normalized 454 sequence count data (N = 755 reactions). A list of the 24 genes can be found in the Figure S2 legend. The analysis reveals a highly significant correlation between the two methods (Pearson's coefficient of correlation  = −0.5614, 95% upper limit  = −0.4558, 95% lower limit  = −0.5614, p<0.0001) that supports the use of normalized 454 counts as a measure of relative gene expression across groups.

**Figure 6:**
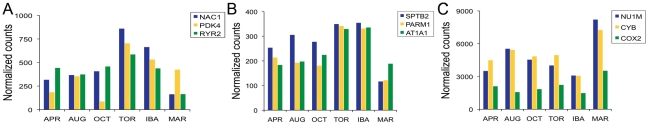
Differentially abundant mRNAs identified in the heart with high sequence read representation in Torpor, Torpor plus IBA or March samples. The colored bars indicate the normalized number of reads at each time point. A. Torpor. NAC1, Sodium/calcium exchanger 1; PDK4, Pyruvate dehydrogenase kinase, isoenzyme 4; RYR2, Ryanodine receptor 2. B. Torpor plus IBA. SPTB2, Spectrin beta chain, brain 1; PARM1, Prostrate androgen-regulated mucin-like protein 1; AT1A1, Sodium/potassium-transporting ATPase subunit alpha-1. C. March. NU1M, NADH-ubiquinone oxidoreductase chain 1; CYB, Cytochrome b; COX2, Cytochrome C Oxidase Subunit 2.

**Figure 7:**
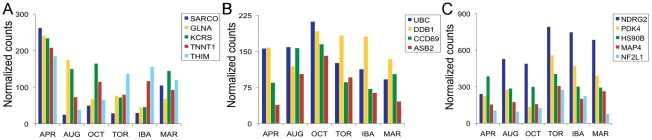
Differentially abundant mRNAs identified in skeletal muscle with high sequence read representation in April, October, or Torpor samples. The colored bars indicate the normalized number of reads at each time point. A. April. SARCO, Sarcolipin; GLNA, Glutamine synthetase; KCRS, Creatine kinase S-type, mitochondrial; TNNT1,Troponin T type 1 slow skeletal muscle; THIM, 3-ketoacyl-CoA thiolase, mitochondrial. B. October. UBC, Ubiquitin C; DDB1, DNA damage-binding protein 1; CCD69, Coiled-coil domain-containing protein 69; ASB2, Ankyrin repeat and SOCS box protein 2. C. Torpor. NDRG2, N-myc downstream regulated gene 2; PDK4, Pyruvate dehydrogenase kinase, isoenzyme 4; HS90B, Heat shock protein HSP 89-beta; MAP4, Microtubule-associated protein 4; NF2L1, Nuclear factor erythroid 2-related factor 1.

**Figure 8:**
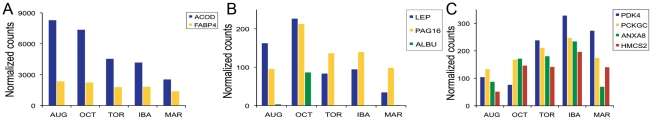
Differentially abundant mRNAs identified in white adipose tissue that had high sequence read representation in August, October, or IBA samples. The colored bars indicate the normalized number of reads at each time point. A. August. ACOD, Acyl Co-A desaturase; FABP4, adipocyte fatty acid binding protein. B. October. LEP, leptin; PAG16, group XVI phospholipase A2; and ALBU, albumin. C. IBA. PDK4, Pyruvate dehydrogenase kinase, isoenzyme 4; PCKGC, phosphoenolpyruvate carboxykinase; ANXA8, annexin A8; HMCS2, hydroxymethylglutaryl-CoA synthase.

Figures 6, 7, 8 highlight some of the genes that met our stringent false discovery rate (FDR) cutoff (1.0×10^−11^) with high mRNA levels at specific time points. The very small FDR was chosen in part to ensure that sufficient numbers of reads were present for reliable identification and assembly of contigs. Our criteria were likely to exclude genes that shared functional relationships but had lower mRNA levels than the genes displayed in Figures 6–8. However, we believe that our approach provides an unbiased picture of the seasonal transcriptome, and that the highlighted genes accurately reflect important changes in the physiology of each tissue. The normalized transcript levels for all genes identified at each of the six time points for all three tissues can be found in Table S1.

The 1.0×10^−11^ FDR criterion resulted in a final set of 155 heart-expressed genes with median mRNA expression of 882 normalized counts (1857, 453; upper and lower quartiles, respectively) across the six samples with Fisher's exact test p-value ≤1.4×10^−13^. In skeletal muscle, 92 genes met our FDR cut off and had a median mRNA expression of 1037 (2180, 510) normalized counts and Fisher's exact test p-value ≤9.6×10^−14^. In WAT, 132 genes met our FDR criteria and had median mRNA expression of 605 (1217, 232) normalized counts across samples and Fisher's exact test p-value ≤6.7×10^−14^. The complete list of expressed genes that meet our FDR criterion for each tissue can be found in Table S2.

### Heart

We highlighted three examples of time point-specific differential gene expression for each of the three tissues. For heart we show highly expressed genes in torpor, torpor and IBA, and March time points (Figure 6A – C). Up-regulated genes during torpor (Figure 6A) included sodium/calcium exchanger 1 (NAC1, 5.3-fold over March), pyruvate dehydrogenase kinase, isoenzyme 4 (PDK4, 8.0-fold over October) and ryanodine receptor 2 (RYR2, 3.5-fold over March).

Three genes that show high expression in heart across both the torpor and IBA time points (Figure 6B) include spectrin beta chain, brain 1 (SPTB2, 3.0-fold over March), prostrate androgen-regulated mucin-like protein 1 (PARM1, 2.7-fold over March), and sodium/potassium-transporting ATPase subunit alpha-1 (AT1A1, 1.8-fold over April). Three mitochondrial genes showing high expression in the heart during March (Figure 6C) included NADH-ubiquinone oxidoreductase chain 1 (NU1M, 2.6-fold over IBA), cytochrome b (CYB, 2.4-fold over IBA), and cytochrome c oxidase subunit 2 (COX2, 2.4-fold over IBA).

### Skeletal muscle

Highly expressed skeletal muscle genes were shown for April, October, and torpor (Figure 7A – C). The five most highly expressed skeletal muscle genes in April (Figure 7A) included sarcopilin (SARCO, 10.5-fold over August), glutamine synthetase (GLNA, 5.4-fold over IBA), mitochondrial S-type creatine kinase (KCRS, 5.1-fold over IBA), slow skeletal muscle troponin T (TNNT1, 2.9-fold over August) and mitochondrial 3-ketoacyl-CoA thiolase (THIM, 4.7-fold over August).

We highlight four highly expressed genes in skeletal muscle during October (Figure 7B). They are polyubiquitin-C (UBC, 2.3-fold over March), DNA damage-binding protein 1 (DDB1, 1.6-fold over August), ankyrin repeat and SOCS box protein 2 (ASB2, 3.6-fold over April) and coiled-coil domain-containing protein 69 (CCD69, 2.3-fold over IBA). Five highly expressed genes from skeletal muscle were highlighted for the torpor timepoint (Figure 7C). These genes included protein NDRG2 (NDRG2, 3.3-fold over April), pyruvate dehydrogenase kinase, isoenzyme 4 (PDK4, 4.1-fold over October), heat shock protein HSP 90-beta (HS90B, 1.4-fold over August), microtubule-associated protein 4 (MAP4, 2.0-fold over April), nuclear factor erythroid 2-related factor 1 (NF2L1, 3.6-fold over March).

### White adipose tissue

Highly expressed genes in WAT were highlighted for the August, October, and IBA time points (Figure 8A – C). We have excluded the April point from our analysis because inclusion of mesenteric fat may have introduced some contamination with pancreatic tissue [7] in the April WAT samples. The genes acyl-coA desaturase (ACOD, 3.2-fold over March) and fatty acid binding protein 4 (FABP4, 1.7-fold over March) were highlighted for WAT in August (Figure 8A). Notably, both ACOD and FABP4 were the most highly expressed genes detected in WAT at any of the six time points.

Three genes expressed highly in WAT during October included leptin (LEP, 6.4-fold over March), albumin (ALBU, at least 21.8-fold over all other points), and group XVI phospholipase A2 (PAG16, 2.2-fold over August), (Figure 8B). Interestingly, expression of albumin was barely detectable at any time point other than October. Genes that were highly expressed during IBA in WAT (Figure 8C) included pyruvate dehydrogenase kinase, isoenzyme 4 (PDK4, 4.3-fold over October), phosphoenolpyruvate carboxykinase (PCKGC, 1.8-fold over August), annexin A8 (ANXA8, 3.4-fold over March), and hydroxymethylglutaryl-CoA synthase (HMCS2, 3.8-fold over August).

## Discussion

In this study we used massive parallel sequencing to determine the identity and level of RNAs involved in mammalian hibernation. In an attempt to paint a comprehensive picture of this circannual phenotype we chose six different time points/activity states (April active, August active, October active, January torpor, January IBA, and March arousal), and selected three tissues (heart, WAT, and skeletal muscle) important for animal survival before, during, and after the hibernation season. Despite the lack of motor activity during torpor skeletal muscle shows minimal effects of disuse atrophy; the heart is a contractile organ that continues to work at near-freezing body temperatures; and WAT serves as the primary supplier of fuel for the entire body in the absence of food.

### Application to other organisms

The Roche 454 sequencing platform was chosen for this study because of relatively long read lengths that can be identified with confidence in the absence of a complete genomic sequence [20]. This is especially true in the case of transcripts that have long and repetitive untranslated regions (UTRs). An example is the 5′-UTR of the gene encoding cold-adapted pancreatic triacylglycerol lipase (LIPP; Figure 3) that contains a retroviral insertion which has highly similar sequence copies elsewhere in the genome [18], [21]. Without a reference genome or transcriptome, it would be impossible to appropriately place such repetitive or widely dispersed sequences with the correct transcript.

Our strategy of deep sequencing the transcriptome can be applied to any non-model organism to uncover vast amounts of information on gene activity where none existed before. Researchers studying plants and animals with novel phenotypes, but lacking the genetic tools associated with popular model organisms, can now generate millions of distinct sequence reads with as little as 1 to 10 µg of total RNA. Computational tools for bioinformatic analysis of these massive data sets are now widely available to researchers around the world. To underscore this point we used only free and open-source software to analyze the sequence data generated in this study; primarily MIRA, Biopython and Sage.

While the intent of this study was to determine differential gene expression between various time points and activity states, deep sequencing revealed a variety of more subtle findings such as transcript splicing variants, gene duplications, heterogeneity in untranslated regions, and a novel method for sequencing the mitochondrial genome. The many benefits of deep sequencing RNA can rapidly increase the amount of molecular information from understudied organisms and therefore enhance the accessibility of these systems to the research community.

### Construction of the mitochondrial genome

Assembly of the mitochondrial genome for *I. tridecemlineatus* was an important byproduct of our transcriptome sequencing (Figures 4 and S1). The traditional approach of sequencing the mitochondrial genome is slow and requires a battery of primers that produce overlapping sequence reads from mitochondrial DNA (mtDNA; [22]). Additionally, mtDNA sequences that reside in the nuclear genome (NuMTs) might complicate or prevent accurate sequencing [23]. Transcriptome sequencing is a quick and accurate method for obtaining a nearly complete mitochondrial genome because only transcribed poly-adenylated RNAs are sequenced. This method of sequencing the mitochondrial genome can be applied to any organism and could rapidly advance our knowledge of species evolution and inform species conservation efforts [24]. Applied to humans, transcriptome sequencing can provide a rapid method of screening for mtDNA mutations and heteroplasmy [25]. Because both nuclear and mitochondrial sequence are obtained simultaneously, the importance of genomic cooperation in species evolution [26] and in health [27] might also be revealed.

### Season and activity-specific gene expression

We highlighted a handful of highly expressed genes from heart, skeletal muscle, and white adipose tissues that are differentially expressed in a time point specific manner (Figures 6, 7, 8). These small sets of genes provide a picture of the seasonal molecular changes that help hibernating mammals survive physiological and environmental extremes. Preparing RNA from animals sacrificed between 9 a.m. and 4 p.m. at each point across the year, combined with the pooling of samples (3 males and 3 females), minimized potential circadian regulation of these genes.

### Heart

Throughout hibernation the heart shows tremendous plasticity during arousal from torpor as heart rate explodes from 5 beats per minute (bpm) to 400 bpm, only to drop back to a state of bradycardia during repeated cycles of torpor and IBA [28]. Ion transport between the cytoplasm and sarcoplasmic reticulum (SR) is crucial for maintenance of heart contraction and relaxation at low body temperatures. Movement of calcium into the SR is required for cardiac muscle relaxation following contraction so that the heart may refill with blood [29]. Two genes that perform this function, RYR2 and NAC1, showed high mRNA levels that peaked in torpor (Figure 6A). Another ion exchanger, AT1A1, is highly expressed during torpor and IBA (Figure 6B). The AT1A1 protein functions to create the electrochemical gradient necessary for heart contraction and also for nutrient transport into the cell [30].

Expression of mitochondrial genes NU1M and CYB, which encode proteins of the electron transport chain, declined from August to torpor and IBA [31]. This likely reflects reduced metabolic rate in the heart during hibernation [28]. When animals are aroused in March and a regular fast heart rate resumes, the transcripts of mitochondrial genes NU1M, CYB, and COX2 are expressed at their highest levels (Figure 6C). Nuclear-encoded mitochondrial protein PDK4 functions to inactivate pyruvate dehydrogenase, preventing the conversion of pyruvate to acetyl-CoA and thus halting the flow of glycolytic intermediates into the tricarboxcylic acid cycle. This blockade to glucose oxidation conserves carbohydrate substrates and dictates the use of lipids as the primary fuel source during hibernation. Deep sequencing showed elevated expression of PDK4 in all three tissues confirming earlier findings involving analysis of RNA blots, protein blots, and expressed sequence tags [16], [32], [33].

### Skeletal muscle

Gene expression reflecting a reduction in motor activity was seen in skeletal muscle. The expression of the structural protein TNNT1 is much lower in August than in April, possibly reflecting the difference in activity level between a recently captured free-living (April) and a captive (August) ground squirrel (Figure 7A). Another indicator of the transition from activity to inactivity is the dramatic decline in abundance of sarcolipin (SARCO) transcripts (Figure 7A). Sarcolipin is a proteolipid that regulates several sarcoplasmic reticulum Ca^2+^-ATPases. In the heart, sarcolipin is regulated by mechanical stress [34] and ablation of the sarcolipin gene results in cardiac hypertrophy [35].

Ubiquitination of proteins is increased in atrophying skeletal muscle (reviewed in [36]). Transcripts coding ubiquitin pathway proteins UBC, ASB2, and DDB1 are abundant and peak in October suggesting that some proteolysis may occur here (Figure 7B). This finding is somewhat counterintuitive because hibernating mammals do not suffer the consequences of muscle atrophy that would normally accompany long-term inactivity [37], [38]. However, Boonstra and colleagues have recently hypothesized that the arctic ground squirrel builds muscle prior to hibernation and then utilizes the muscle during winter as a fuel source in addition to white adipose tissue [39]. Elevated expression of ubiquitin pathway proteins involved in proteolysis supports this hypothesis and offers a potential mechanism for extracting nutrients from skeletal muscle.

In skeletal muscle n-Myc downstream regulated gene 2 (NDRG2) and PDK4 are most highly expressed during torpor, IBA, and March (Figure 7C). NDRG2 has been shown to be expressed in heart tissue in response to ischemia/reperfusion injury and is thought to be protective against apoptosis [40]. Increased expression of HS90B, MAP4, and NF2L1 during torpor also indicate that cell and genome protective mechanisms are upregulated during this period [41].

### White adipose

The annual natural history of WAT in a hibernating mammal can be divided into lipogenic and lipolytic periods that are reflected in the relative abundance of mRNAs encoding lipogenic or lipolytic proteins [42]. The August and October samples represent the lipogenic period while the torpor, IBA, and March samples represent the lipolytic period.

In this study we observed a high abundance of three lipogenesis-related genes in the August and October samples (Figure 8A-B). ACOD functions to insert a double bond into a wide range of fatty acyl-CoA substrates [43]. Adipocyte fatty acid binding protein FABP4 is one of two fatty acid binding proteins that are specifically found in the cytosol of adipocytes, the other is FABP5. FABP4 has a high affinity for binding long-chain fatty acids and retinoic acid and is thought to function primarily in intracellular fatty acid transport. A role for FABP4 in regulating cellular lipid metabolism is suggested from observed interactions with two important regulators of lipid homeostasis, hormone sensitive lipase (HSL;[44]) - the rate limiting enzyme in triglyceride metabolism [45]; and peroxisome proliferator activated receptor-gamma (PPAR-γ) [46], [47] - a molecule that regulates the balance of lipogenesis and lipolysis [48]. The FABP4 protein is considered to be a marker of lipid accumulation, obesity, and inflammation in morbidly obese women [49] and is also known as adipocyte protein 2 (aP2). FABP4 is a target for drug development in the treatment of atherosclerosis, diabetes, and asthma [50], [51].

The mRNA encoding leptin was observed to have greatest abundance during the August and October lipogenic phase (Figure 8B). This is not a surprising result since leptin and its mRNA are known to increase in abundance with increasing fat mass [52]. Leptin mRNA continues to be highly abundant throughout the lipogenic period suggesting the development of leptin resistance. In rodents, leptin resistance is associated with hyperphagia, increased lipogenesis and feed efficiency, and decreased core body temperature and physical activity [53]. Townsend and colleagues have proposed that the effects of leptin resistance may prepare a hibernating mammal for torpor [54].

The large increase in albumin expression during October (Figure 8B) may indicate preparation for, or initiation of, the seasonal transition to lipolysis. Albumin is a major component of blood serum and functions to transport endogenous compounds through the blood, including nonesterified or free fatty acids (FFA; [55]). A high level of albumin expression in October would prepare the squirrel for mobilization of lipids from storage in white adipose tissue, and through the blood to target tissues. Albumin carries both medium- and long-chain and mono- and polyunsaturated fatty acids [56]. The human albumin protein has at least seven binding sites for FFA with the normal FFA to albumin ratio in the range of 0.1-2∶1, and during fasting the ratio can rise to 6∶1 [57].

### Summary

Deep sequencing of the transcriptome as a means to interrogate the molecular basis of the hibernation phenotype has revealed changes in the expression of specific genes at various times throughout the year. This new approach of measuring gene activity opens the door for investigating other novel phenotypes in the vast world of “non-model” organisms. In thirteen-lined ground squirrels we not only found new patterns of gene activity in heart, skeletal muscle and white adipose, we also generated sequence for over 14,000 different mRNAs and used a portion of the transcriptome data to generate the complete sequence of the mitochondrial genome. As expected we found differentially expressed genes involved in the carbohydrate to lipid fuel switch, as well as enhanced ion transport required for heart contraction during torpor. Interestingly, transcripts coding ubiquitin pathway proteins UBC, ASB2 and DDB1 are abundant and peak in October. This intriguing finding suggests that ubiquitin-mediated proteolysis could provide a mechanism by which skeletal muscle can be catabolized and serve as a secondary fuel depot during winter.

The overall abundance of genes with varying expression patterns in the entire dataset presents an unparalleled opportunity to explore the molecular mechanisms controlling hibernation in mammals. The normalized transcript levels for all genes identified at each of the six time points from all three tissues can be found in the supporting information (Table S1) and is therefore available to the greater research community for further analysis.

## Supporting Information

Figure S1 Mitochondrial genome sequence for *I. tridecemlineatus* constructed from RNA sequence reads.
(TXT)

Figure S2 Correlation between qRT-PCR and normalized RNA sequence read counts for 24 different mRNAs expressed in WAT isolated from individual animals (N = 6) at five time points from August through March. Log transformed qRT-PCR “Take off CT” values (24 transcripts, 30 individual animals, 755 reactions) was correlated with log transformed normalized 454 counts for pooled samples. The analysis reveals a significant correlation between methods (Pearson's coefficient of correlation  = −0.5614, 95% upper limit  = −0.4558, 95% lower limit  = −0.5614, p<0.0001) that supports the use of normalized 454 counts as a measure of relative mRNA expression across groups. *Methods -* A cycle number which is 20% of the second derivative maximum of the amplification plot for qRT-PCR reactions, defined in the Rotor-gene 3000 software as “Take off CT”, was used as an objective measure of crossing threshold (CT) for comparison between qRT-PCR reactions. The mRNAs that were measured coded for proteins with the following human UniProt abbreviations: ACDSB, AKAP1, CCDC50, CELF2, CYCA, HIF1N, HNRL1, INMT, LAMP1, LIPP, MMSA, MPRIP, MYH9, PDK4, PLEC1, PTGER3, PTGR1, RN115, RPB2, SLCO2B1, SRPK1, TM63B, TUSC5, ZEB1.
(DOC)

Table S1 Normalized transcript levels for all genes identified at each of the six time points for heart, skeletal muscle, and WAT.
(XLS)

Table S2 Complete list of expressed genes that meet the 1.0×10^−11^ FDR criterion for heart, skeletal muscle, and WAT.
(XLS)
