# Supplementary material for: Deep Sequencing the Transcriptome Reveals Seasonal Adaptive Mechanisms in a Hibernating Mammal
Source: PLoS One. 2011 Oct 28;6(10):e27021. doi: 10.1371/journal.pone.0027021 (PMC3203946; doi:10.1371/journal.pone.0027021)

Correlation between qPCR Take off CT values and Normalized 454 counts

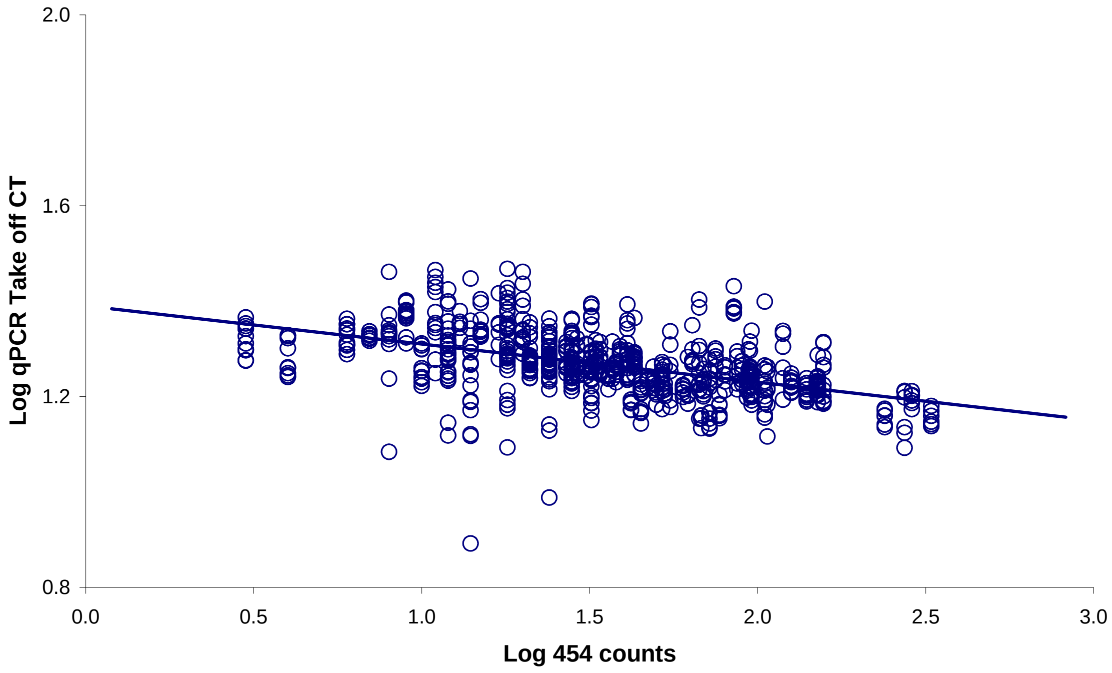

Supplement: Supplementary file 1 [file pone.0027021.s002.doc]
